# von Willebrand factor Ristocetin co-factor activity to von Willebrand factor antigen level ratio for diagnosis of acquired von Willebrand syndrome caused by aortic stenosis

**DOI:** 10.1016/j.rpth.2023.102284

**Published:** 2023-11-30

**Authors:** Noriyuki Okubo, Shingo Sugawara, Tohru Fujiwara, Ko Sakatsume, Tsuyoshi Doman, Mihoko Yamashita, Kota Goto, Masaki Tateishi, Misako Suzuki, Ryutaro Shirakawa, Yuka Eura, Koichi Kokame, Masaki Hayakawa, Masanori Matsumoto, Yasunori Kawate, Mizuki Miura, Hiroshi Takiguchi, Yoshimitsu Soga, Shinichi Shirai, Kenji Ando, Yoshio Arai, Takaharu Nakayoshi, Yoshihiro Fukumoto, Hiroyuki Takahama, Satoshi Yasuda, Toshihiro Tamura, Shin Watanabe, Takeshi Kimura, Nobuhiro Yaoita, Hiroaki Shimokawa, Yoshikatsu Saiki, Koichi Kaikita, Kenichi Tsujita, Shinji Yoshii, Hiroshi Nakase, Shin-ichi Fujimaki, Hisanori Horiuchi

**Affiliations:** 1 Department of Clinical Laboratory Medicine, Tohoku University Hospital, Sendai, Japan; 2 Department of Hematology, Tohoku University Graduate School of Medicine, Sendai, Japan; 3 Department of Molecular and Cellular Biology, Institute of Development, Aging and Cancer, Tohoku University Graduate School of Medicine, Sendai, Japan; 4 Division of Cardiovascular Surgery, Tohoku University Graduate School of Medicine, Sendai, Japan; 5 Department of Molecular Pathogenesis, National Cerebral and Cardiovascular Center, Suita, Japan; 6 Department of Blood Transfusion Medicine, Nara Medical University, Kashihara, Japan; 7 Medical Affairs 2, Medical & Scientific Affairs, Sysmex Corporation, Kobe, Japan; 8 Department of Cardiology, Kokura Memorial Hospital, Kokura-kitaku, Kitakyushu, Japan; 9 Department of Cardiovascular Surgery, Kokura Memorial Hospital, Kokura-kitaku, Kitakyushu, Japan; 10 Division of Cardiovascular Medicine, Department of Internal Medicine, Kurume University School of Medicine, Kurume, Japan; 11 Cardiovascular Department, National Cerebral and Cardiovascular Center, Suita, Japan; 12 Department of Cardiovascular Medicine, Tohoku University Graduate School of Medicine, Sendai, Japan; 13 Department of Cardiology, Tenri Hospital, Tenri, Japan; 14 Department of Cardiovascular Medicine, Graduate School of Medicine, Kyoto University, Kyoto, Japan; 15 Department of Cardiovascular Medicine, Graduate School of Medical Sciences, Center for Metabolic Regulation of Healthy Aging, Kumamoto University, Kumamoto, Japan; 16 Department of Gastroenterology and Hepatology, Sapporo Medical University School of Medicine, Sapporo, Japan

**Keywords:** aortic stenosis, acquired von Willebrand syndrome, von Willebrand factor, VWF Ristocetin co-factor activity, VWF:RCo/VWF:Ag

## Abstract

**Background:**

Severe aortic stenosis (AS) causes acquired von Willebrand syndrome by the excessive shear stress–dependent cleavage of high molecular weight multimers of von Willebrand factor (VWF). While the current standard diagnostic method is so-called VWF multimer analysis that is western blotting under nonreducing conditions, it remains unclear whether a ratio of VWF Ristocetin co-factor activity (VWF:RCo) to VWF antigen levels (VWF:Ag) of <0.7, which can be measured with an automated coagulation analyzer in clinical laboratories and is used for the diagnosis of hereditary von Willebrand disease.

**Objectives:**

To evaluated whether the VWF:RCo/VWF:Ag is useful for the diagnosis of AS-induced acquired von Willebrand syndrome.

**Methods:**

VWF:RCo and VWF:Ag were evaluated with the VWF large multimer index as a reference, which represents the percentage of a patient’s VWF high molecular weight multimer ratio to that of standard plasma in the VWF multimer analysis.

**Results:**

We analyzed 382 patients with AS having transaortic valve maximal pressure gradients of >30 mmHg, 27 patients with peripheral artery disease, and 46 control patients free of cardiovascular disease with osteoarthritis, diabetes, and so on. We assumed a large multimer index of <80% as loss of VWF large multimers since 59.0% of patients with severe AS had the indices of <80%, while no control patients or patients with peripheral artery disease, except for 2 patients, exhibited the indices of <80%. The VWF:RCo/VWF:Ag ratios, measured using an automated blood coagulation analyzer, were correlated with the indices (r_s_ = 0.470, *P* < .001). When the ratio of <0.7 was used as a cut-off point, the sensitivity and specificity to VWF large multimer indices of <80% were 0.437 and 0.826, respectively.

**Conclusion:**

VWF:RCo/VWF:Ag ratios of <0.7 may indicate loss of VWF large multimers with high specificity, but low sensitivity. VWF:RCo/VWF:Ag ratios in patients with AS having a ratio of <0.7 may be useful for monitoring the loss of VWF large multimers during their clinical courses.

## Essentials

• von Willebrand factor Ristocetin co-factor activity to von Willebrand factor antigen level ratio (VWF:RCo/VWF:Ag) classifies von Willebrand disease.
• Aortic stenosis can cause acquired von Willebrand syndrome via loss of von Willebrand factor (VWF) large multimers.
• We found that VWF:RCo/VWF:Ag ratio inversely correlated with large multimer loss in aortic stenosis.
• VWF:RCo/VWF:Ag ratio of <0.7 indicated loss of VWF large multimers with high specificity.

## 1 Introduction

Vascular endothelial cells and megakaryocytes produce and secrete von Willebrand factor (VWF) as giant multimers [1,2]. VWF multimers are subsequently cleaved by a specific cleaving enzyme, a disintegrin and metalloproteinase with a thrombospondin type 1 motif, member 13 (ADAMTS-13), in a shear stress–dependent manner. Thus, VWF is present in plasma as multimers composed of fewer than 80 subunits. Of VWF multimers, it is well known that those of higher molecular weight play important roles in hemostasis [3,4]. Therefore, a genetic disorder that causes the loss of VWF high molecular weight multimers leads to a hemostatic disorder classified as von Willebrand disease type 2A [5]. Such a loss of VWF high molecular weight multimers secondarily occurs in some cardiovascular diseases such as aortic stenosis (AS) and is designated as acquired von Willebrand syndrome (AVWS). In patients with these cardiovascular diseases excessive high shear stress is generated in the bloodstream, where the cleavage of high molecular weight VWF multimers is enhanced. AS, a condition of increased shear stress across the stenotic aortic valve, is one cause of such AVWS [6] and is sometimes accompanied by gastrointestinal bleeding, known as Heyde syndrome [[7], [8], [9]].

The current standard diagnostic method for shear stress–induced AVWS is the VWF multimer analysis, which consists of sodium dodecyl sulfate–agarose gel electrophoresis under nonreducing conditions followed by western blotting. Conventionally, bands higher than the 10th from the lowest molecular weight band in the analysis have been defined as high molecular weight (large) multimers. Although the AVWS is characterized by a loss of high molecular weight multimers, a clear definition of AVWS or its severity classification do not exist, since quantitative evaluation methods for the VWF multimer analysis have not been established to date. We proposed a quantitative value termed as the “VWF large multimer index” [6,10,11], which has been widely used recently [12,13]. The index is defined as the ratio of the patient’s VWF large multimer ratio to that of control. We showed that the indices inversely correlated with the severity of AS evaluated by transaortic valve maximal pressure gradients [10]. We also showed that the loss of VWF large multimers, evaluated by the index, in patients under mechanical circulatory supports, such as venoarterial extracorporeal membrane oxygenation [10] and an implantable left ventricular assist device [11], is more severe than that in patients with severe AS.

The VWF multimer analysis consists of many subtle technical procedures since the molecular weights of the largest VWF multimers are extraordinarily high at around 20,000 kDa and it takes 2 to 3 days as the shortest time. In comparison, for the diagnosis of hereditary von Willebrand disease type 2A, the ratio of VWF Ristocetin co-factor activity (VWF:RCo) to the VWF antigen level (VWF:Ag) of less than 0.7 [5,[14], [15], [16]] or 0.6 [17,18] is used for the diagnosis. It has been reported that VWF:RCo/VWF:Ag of <0.7 indicates the disease with a sensitivity of 92% and a specificity of 72% [19]. Thus, in a widely used guideline, VWF:RCo/VWF:Ag of <0.7 is used as a marker of von Willebrand disease type 2A [20]. Since both VWF:RCo and VWF:Ag can be feasibly measured with an automated coagulation analyzer in clinical laboratories, it would be useful in clinical settings if AVWS caused by AS can be diagnosed using VWF:RCo/VWF:Ag. Nevertheless, VWF:RCo/VWF:Ag has not been evaluated as a diagnostic value of AVWS in studies with a large number of patients with AS.

To evaluate whether the VWF:RCo/VWF:Ag is useful for the diagnosis, we analyzed this ratio in a large number of patients with severe AS (*n* = 382) and evaluated the ratios in comparison with VWF large multimer indices.

## 2 Methods

### 2.1 Study population

This study was a substudy of the AVWS co-existing with cardiovascular diseases study (the AVeC study) (UMIN000018135), a multi-institutional observational study. In the study, patients with various cardiovascular diseases were enrolled between September 2015 and February 2021. Of these, patients with AS and peripheral artery disease (PAD) enrolled by July 2020 were analyzed. We also analyzed residual plasmas in the Clinical Laboratory, Tohoku University Hospital, from patients with the following criteria: absence of cardiovascular disease, age of >70 years, hemoglobin levels of >10 mg/dL, creatinine levels of <1.5 mg/dL, total bilirubin levels of <2.5 mg/dL, and albumin levels of >3.0 g/dL. These data were evaluated in the same way as for the control patients.

### 2.2 Laboratory data collection

Blood from each study participant was collected in a 3.13% citrate-containing tube and centrifuged to obtain plasma. Plasma samples were frozen and transferred to the laboratory in the Institute of Development, Aging and Cancer, Tohoku University, Sendai City, Japan, where they were kept in a deep freezer at −80 ^°^C until analysis.

VWF:RCo (BC Von Willebrand Reagent, Siemens Healthcare Diagnostics), VWF:Ag (vWF Ag reagent, Siemens Healthcare Diagnostics), and activity of a coagulation factor, factor (F) VIII (FVIII) (Revohem FVIII Chromogenic, Sysmex), were measured using an automated coagulation analyzer, CS-5100 (Sysmex), according to the manufacturer’s instructions. Since measurements of FVIII activity were initiated in the middle of the study, all samples were not measured for this and a part of the samples (*n* = 123) was analyzed. Standard human plasma (Siemens Healthcare Diagnostics) was used for calibration.

**Figure 1:**
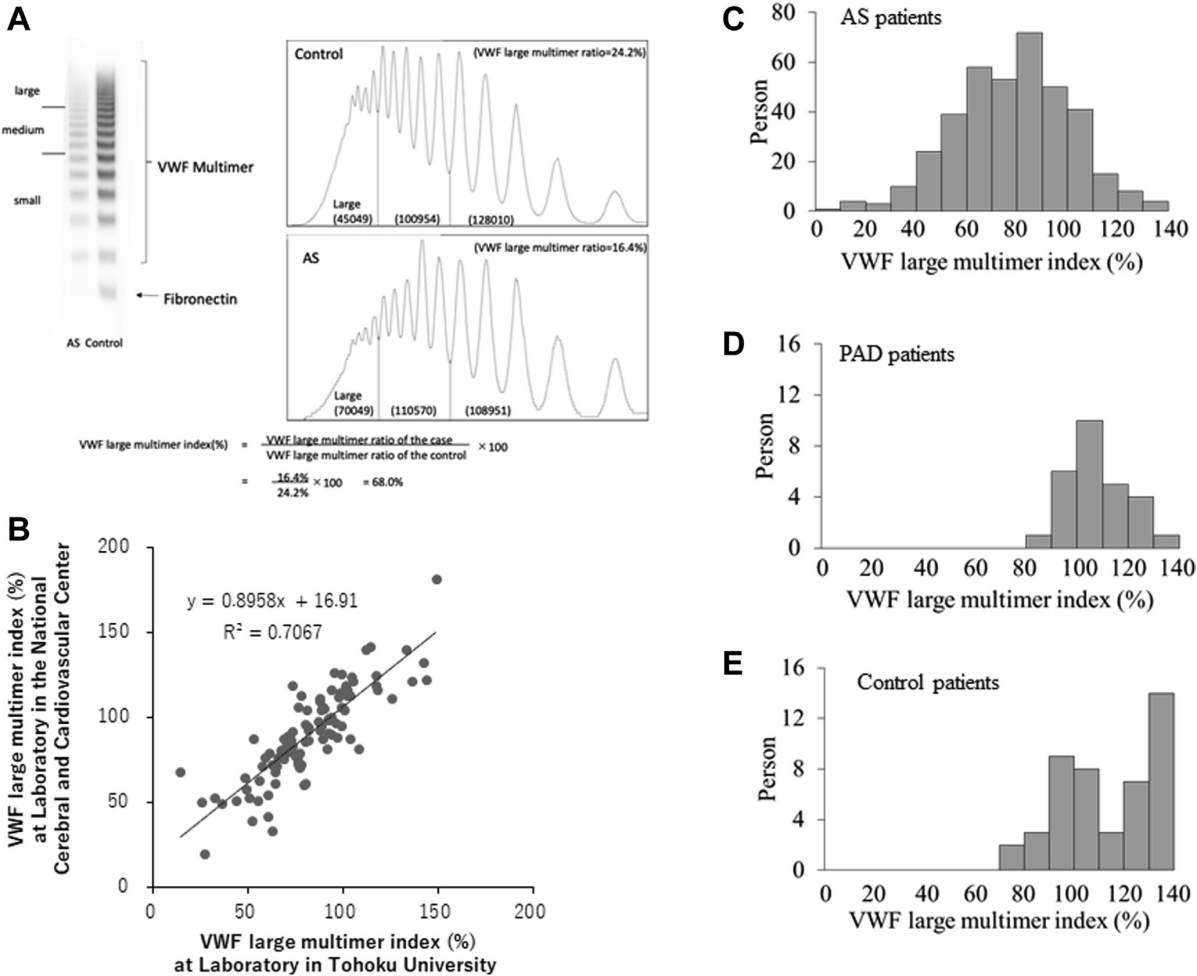
Distribution of the VWF large multimer indices. (A) The formula for calculating the VWF large multimer index [6,10,11] is shown. The 11th and higher bands from the lowest were classified as large multimers (high molecular weight multimers). Based on densitometric analysis, the index was calculated as a percentage of a patient's high molecular weight VWF multimer ratio to that of the control (the Siemens Standard plasma) analyzed in a lane adjacent to that of the patient's plasma. An example is shown in this figure. It is noted that the left lane with a sample of an aortic stenosis patient’s plasma exhibited a VWF large multimer index of 68.0%. (B) Of enrolled patients’ plasmas, 105 samples were randomly selected and VWF multimer indices were measured in 2 independent laboratories using the same primary antibody. (C–E) Histograms of VWF large multimer indices for samples from patients with aortic stenosis (C), or peripheral artery disease (D), and control patients (E) are shown. It is noted that neither peripheral artery disease nor control patients except 2 patients showed the indices of <80%. VWF, von Willebrand factor.

The VWF multimer analysis was performed by electrophoresis under nonreducing conditions using a 1.0% or 1.2% agarose gel, where equal amounts of VWF:Ag were loaded in each lane, which was subsequently followed by western blotting using an anti-VWF antibody (DAKO) as the primary antibody. Bands over the 10th from the lowest were classified as large multimers (high molecular weight multimers). Based on densitometric analysis, the VWF large multimer index was calculated as the percentage of a patient’s high molecular weight VWF multimer ratio to that of control (the Siemens Standard plasma) analyzed in the adjacent lane of the patient’s plasma (Figure 1A), as described previously [6,10,11]. We have shown that the lowest band is not VWF but fibronectin, due to the cross-reactivity of anti-VWF antibody [21]. Thus, we measured the VWF multimer indices by analyzing the VWF multimer bands without the fibronectin band as shown in Figure 1A. To examine reproducibility of the method, 105 plasma samples from enrolled patients were independently analyzed by the National Cerebral and Cardiovascular Center and by Tohoku University using the same primary antibody. As shown in Figure 1B, the VWF multimer indices measured by both laboratories correlated well, indicating that the indices could provide reproducible results beyond laboratories.

### 2.3 Statistical analysis

Statistical analyses were performed with IBM SPSS statistics version 22 (IBM). Continuous variables were expressed as mean ± SD or median (IQR). Categorical variables were presented as frequencies and percentages. Correlations between variables were assessed by the Spearman’s rank correlation test. The Kruskal–Wallis test was used for multiple comparisons between groups. All statistical analyses were considered significant with *P* < .05.

### 2.4 Ethics

The study was approved by the ethics committees of all participating institutions. Written informed consent was obtained from each patient. This study was conducted in accordance with the Declaration of Helsinki.

## 3 Results

### 3.1 Patient characteristics

In the study, 468 patients with AS with a peak pressure gradient through the aortic valve of >30 mmHg, as determined by Doppler echocardiography, were enrolled. Some patients exhibited VWF:RCo or VWF:Ag of >300% that was the upper limit of the measurement of both values in the automated coagulation analyzer used in this study. In such cases, we could measure them by dilution of their plasmas in some patients, while we could not in others due to the limitation in the volumes of stored plasmas. In this study, 86 patients were excluded mainly due to missing the data that were required for this study. Thus, 382 patients with AS were analyzed. In addition, 27 patients with PAD hospitalized for angioplasty, and 46 control patients were analyzed.

Patient characteristics are shown in Table 1. Patients with AS, patients with PAD, and control patients had mean ages of 82 ± 7 (mean ± SD), 73 ± 9, and 76 ± 5 years, respectively. The 382 patients with severe AS analyzed in this study had a maximal pressure gradient through the aortic valve of 84.6 ± 32.9 mmHg (mean ± SD) and an aortic valve area of 0.70 ± 0.18 cm^2^. Surgical aortic valve replacement and transcatheter aortic valve implantation were performed for 105 patients (27.5%) and 220 (57.6%) patients, respectively. All 27 enrolled patients with PAD underwent angioplasty.

**Table 1:** Characteristics of participants.

	Patients with AS (*n* = 382)	Patients with PAD (*n* = 27)	Control patients (*n* = 46)	*P* value	
				Patients with AS vs patients with PAD	Patients with AS vs control patients
Age (y)	82 ± 7	73 ± 9	76 ± 5		
Sex				<.001	.51
Male	127 (33.2)	19 (70.4)	18 (39.1)		
Race/ethnicity					
Asian	382 (100.0)	27 (100.0)	46 (100.0)		
ABO blood type				<.001	.52
O	108 (28.3)	0 (0.0)	17 (37.0)		
Non-O	269 (70.4)	0 (0.0)	27 (58.7)		
Unknown	5 (1.3)	27 (100.0)	2 (4.3)		
Hemoglobin (g/dL)	11.4 ± 1.8	12 ± 2	12.8 ± 1.7		
Platelet (×10^9^/L)	185 ± 63	49 ± 12	218 ± 50		
D-dimer (μg/mL)	2.0 ± 2.9	2.1 ± 2.9	NT		
FVIII (%)	116.4 ± 46.1	69.4 ± 24.8	NT		
VWF:Ag (%)	168.4 ± 53.1	119.2 ± 38.6	182.9 ± 56.2	<.001	.103
VWF:RCo (%)	131.0 ± 50.9	96.1 ± 35.8	167.8 ± 49.8	.005	<.001
VWF:RCo/VWF:Ag	0.77 ± 0.15	0.81 ± 0.16	0.92 ± 0.11	.22	<.001
VWF large multimer index (%)	78.2 ± 22.8	108.4 ± 12.0	115.0 ± 24.0	<.001	<.001
Maximal aortic gradient (mmHg)	84.6 ± 32.9	NT	NT		
Aortic valve area (cm^2^)	0.70 ± 0.18	NT	NT		

Values are mean ± SD or *n* (%).

AS, aortic stenosis; PAD, peripheral artery disease; NT, not tested; FVIII, factor VIII; VWF, von Willebrand factor; VWF:Ag, von Willebrand factor antigen; VWF:RCo, von Willebrand factor Ristocetin co-factor.

The VWF-related values in patients with AS and each blood type are shown in Table 2. VWF:Ag (*P* < .001) and VWF:RCo (*P* < .001) of patients with blood type O were significantly lower than the others. On the other hand, VWF:RCo/VWF:Ag or VWF large multimer indices were not significantly different between patients with blood type O and the others (*P* = .23 and *P* = .08, respectively).

**Table 2:** VWF-related value for ABO blood type (type O vs non-O) in patients with aortic stenosis.

	ABO Blood type		*P* value
	O (*n* = 108)	Non-O (*n* = 269)	
VWF:Ag (%)	140.8 ± 46.3	178.5 ± 51.7	<.001
VWF:RCo (%)	107.2 ± 42.9	139.7 ± 50.9	<.001
VWF:RCo/VWF:Ag	0.76 ± 0.15	0.78 ± 0.15	.23
VWF large multimer index (%)	81.2 ± 23.4	76.6 ± 22.4	.08

Values are mean ± SD.

VWF, von Willebrand factor; VWF:Ag, von Willebrand factor antigen; VWF:RCo, von Willebrand factor Ristocetin co-factor.

### 3.2 VWF large multimer indices <80% may indicate AVWS in patients with AS

The VWF large multimer indices of patients with AS, patients with PAD, and control patients were 78.2 ± 22.8 % (mean ± SD), 108.4 ± 12.0 %, and 115.0 ± 24.0%, respectively. Indices of <80% exhibited an apparent reduction in the large multimers (Figure 1A). Importantly, 59.0% of patients with severe AS and transaortic valve peak pressure gradients ≥64 mmHg (the maximal velocity >4.0 m/sec) had the indices of <80%, while no patients with PAD or control patients except 2 patients showed the indices of <80% (Figure 1C–E). Thus, we here defined that the VWF large multimer indices of <80% indicate loss of VWF large multimers.

### 3.3 Relationship between VWF:RCo/VWF:Ag and VWF large multimer indices

**Figure 2:**
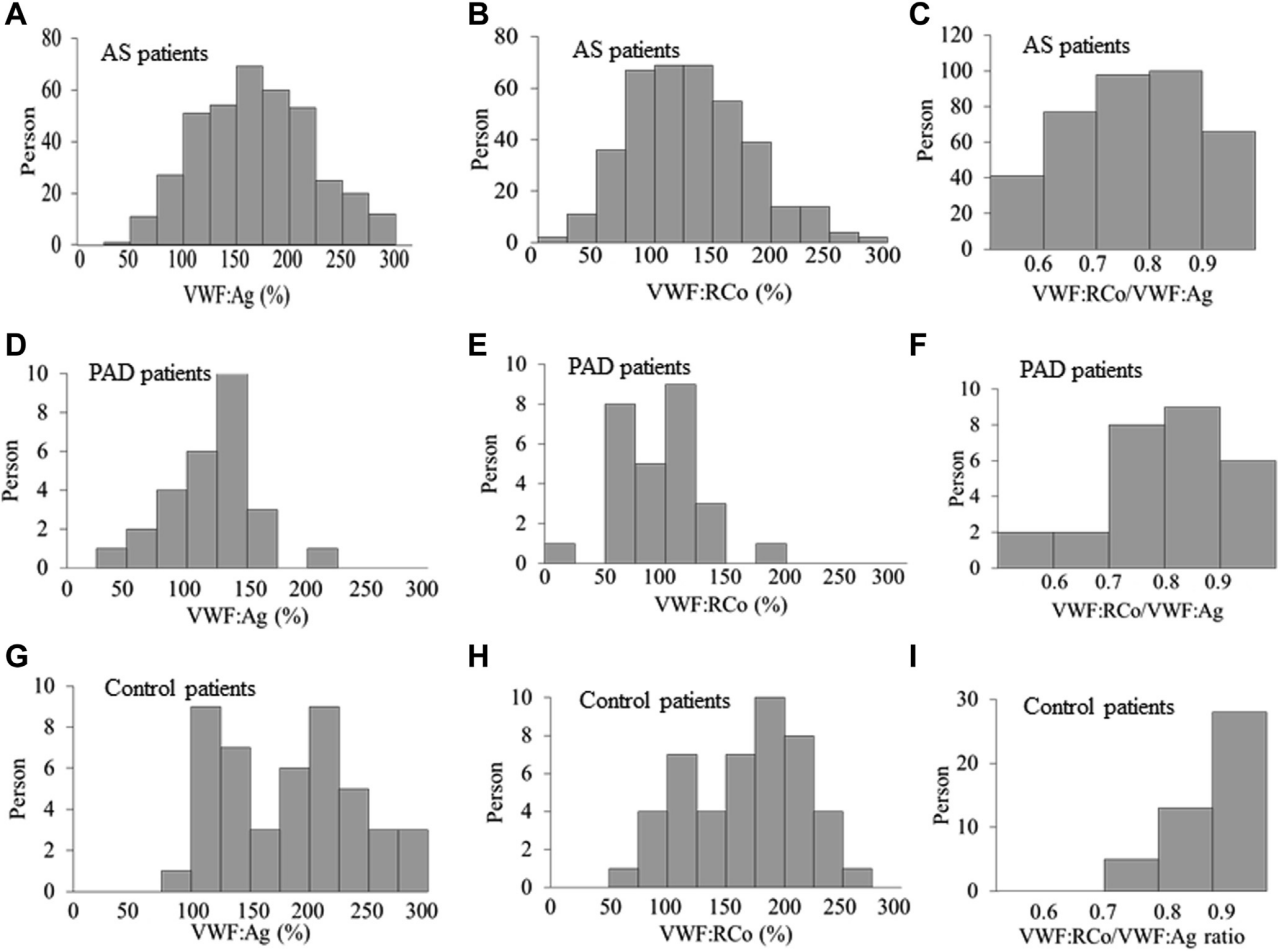
Histograms of VWF:Ag (A, D, G), VWF:RCo (B, E, H), and VWF:RCo/VWF:Ag (C, F, I) in patients with AS (A–C) or PAD patients (D–F), and control patients (G–I).

**Figure 3:**
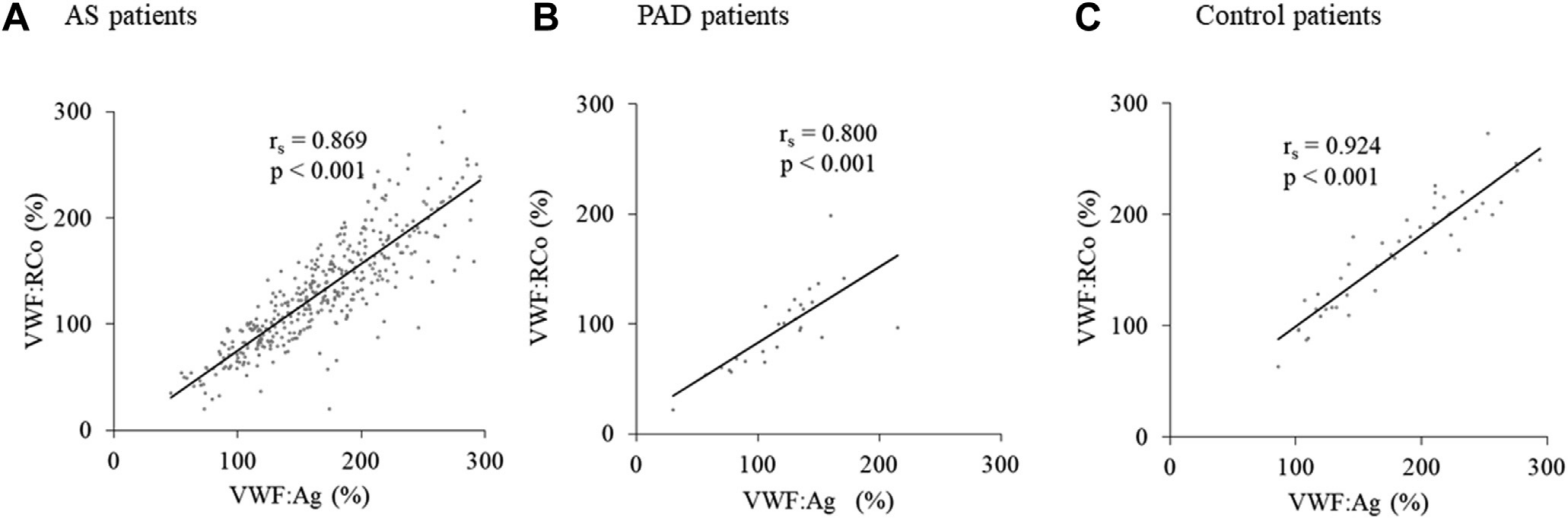
Correlation of von Willebrand factor antigen with von Willebrand factor Ristocetin co-factor activity in patients with aortic stenosis (A) or peripheral artery disease (B), and control patients (C).

Patients with AS exhibited higher VWF:Ag and VWF:RCo levels than patients with PAD (compare Figure 2A with 2D and Figure 2B with 2E, respectively). There were no statistical significance in VWF:Ag between patients with AS and control patients (*P* = .103), while VWF:RCo of patients with AS was lower than that of control patients (*P* < .001). Since VWF:RCo levels were closely correlated with VWF:Ag levels in patients with AS (r_s_ = 0.869, *P* < .001), patients with PAD (r_s_ = 0.800, *P* < .001), and control patients (r_s_ = 0.924, *P* < .001) (Figure 3), the high VWF:RCo levels often observed in patients with AS could be due to rather high VWF:Ag levels.

**Figure 4:**
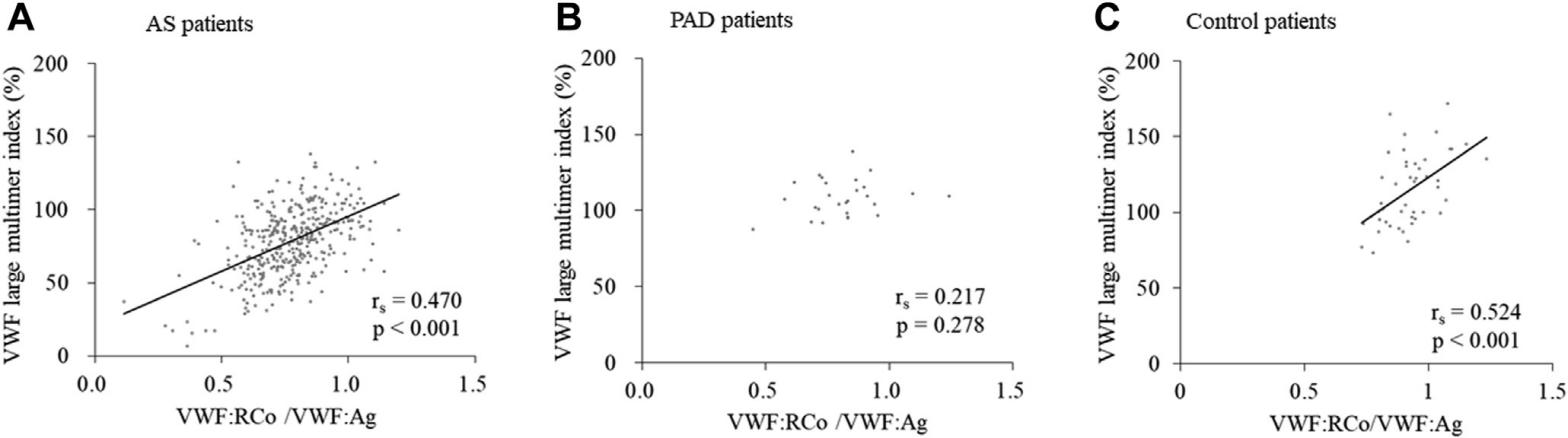
Correlation of von Willebrand factor Ristocetin co-factor activity/von Willebrand factor antigen with von Willebrand factor large multimer indices. (A) In patients with aortic stenosis (r_s_ = 0.470, *P* < .001), (B) or peripheral artery disease (r_s_ = 0.217, *P* = .28), and (C) control patients (r_s_ = 0.524, *P* < .001).

VWF:RCo/VWF:Ag of patients with AS was significantly lower than those of control patients (*P* < .001), while there were no significance in VWF:RCo/VWF:Ag between patients with AS and patients with PAD (*P* = .22) (Table 1). In comparison, 30.9% of patients with AS showed VWF:RCo/VWF:Ag of <0.7, while for patients with PAD and control patients, it was 14.8% (AS vs PAD; *P* = .09) and 0.0% (patients with AS vs control patients; *P* < .001), respectively (Figure 2C, F, and I). Furthermore, VWF:RCo/VWF:Ag ratios were positively correlated with VWF large multimer indices in patients with AS (r_s_ = 0.470, *P* < .001), and in control patients (r_s_ = 0.542, *P* < .001), while they did not correlate in patients with PAD (r_s_ = 0.217, *P* = .28) (Figure 4).

**Figure 5:**
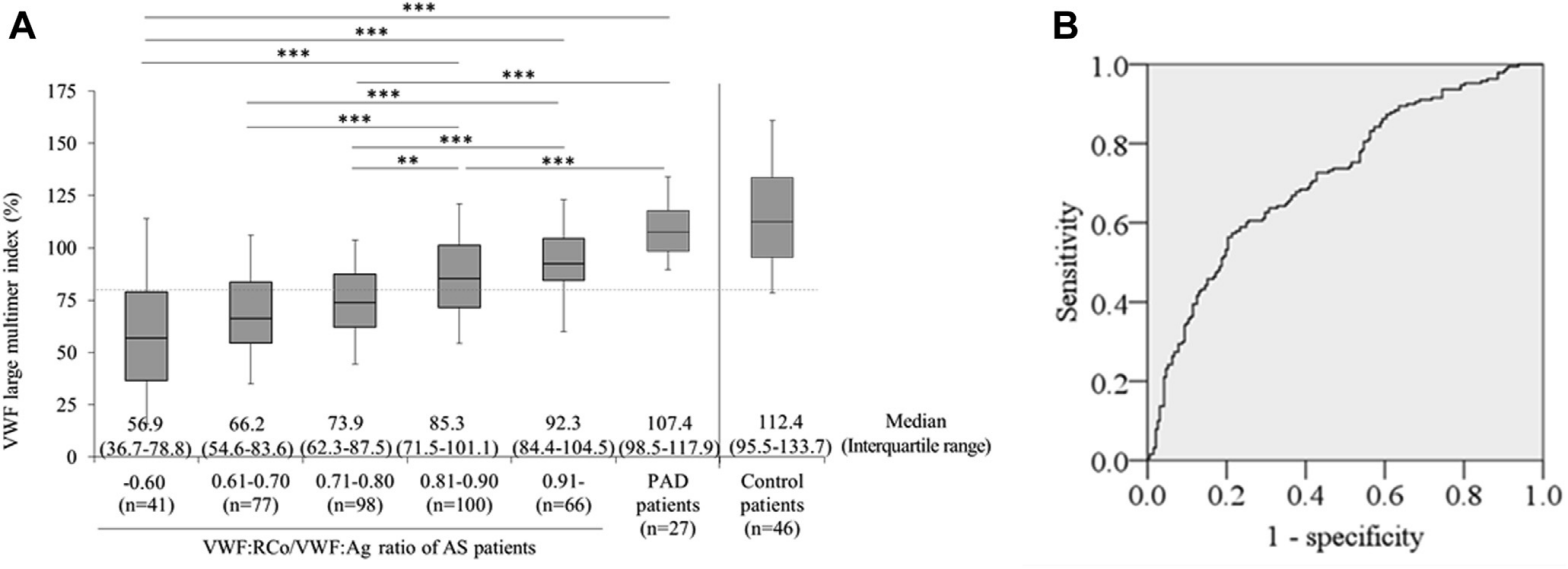
Relationship between VWF large multimer indices and VWF:RCo/VWF:Ag. (A) The VWF large multimer indices according to the ranges of the VWF:RCo/VWF:Ag values in patients with aortic stenosis. Those of patients with PAD and the control patients are also shown. The dotted line indicates a VWF large multimer index of 80.0%. Median VWF large multimer indices for groups with VWF:RCo/VWF:Ag of ≤0.60, 0.61-0.70, 0.71-0.80, 0.81-0.90, and >0.9 groups are 56.9% (IQR: 36.7%-78.8%), 66.2% (54.6%-83.6%), 73.9% (62.3%-87.5%), 85.3% (71.5%-101.1%), and 92.3% (84.4%-104.5%), respectively. Asterisks indicate significant differences (Kruskal–Wallis test, ∗∗*P* < .01, ∗∗∗*P* < .001). (B) The receiver operating characteristic curve for VWF:RCo/VWF:Ag for the loss of VWF large multimer indices (<80.0%). When the VWF:RCo/VWF:Ag of <0.7 was used as a cut-off point, the sensitivity and specificity for the loss of VWF large multimers were 0.437 and 0.826, respectively (area under the curve: 0.717, 95% CI: 0.67-0.77). VWF, von Willebrand factor; VWF:Ag, VWF antigen; VWF:RCo, VWF Ristocetin co-factor activity.

We then analyzed the data of VWF large multimer indices (Figure 4) by stratifying VWF:RCo/VWF:Ag. The indices increased in a VWF:RCo/VWF:Ag-dependent manner (Figure 5A). The median VWF large multimer indices for VWF:RCo/VWF:Ag ≤0.60, 0.61-0.70, 0.71-0.80, 0.81-0.90, and >0.9 groups were 56.9% (IQR: 36.7%-78.8%), 66.2% (54.6%-83.6%), 73.9% (62.3%-87.5%), 85.3% (71.5%-101.1%), and 92.3% (84.4%-104.5%), respectively. The percentages of patients showing the VWF large multimer indices <80% were 78.0% (32 in 41), 67.5% (52 in 77), 58.1% (57 in 98), 37.0% (37 in 100), and 21.2% (14 in 66), respectively. Thus, 71.2% (84 in 118) of patients with VWF:RCo/VWF:Ag of <0.7 showed the VWF large multimer indices of <80%.

The receiver operating characteristic curve for VWF large multimer indices and VWF:RCo/VWF:Ag is shown in Figure 5B. When the VWF:RCo/VWF:Ag ratio of <0.7 was used as a cut-off point, the sensitivity and specificity for the loss of VWF large multimers (VWF large multimer indices <80%) were 0.437 and 0.826, respectively (area under the curve: 0.717, 95% CI: 0.67-0.77).

### 3.4 Correlation of VWF:RCo/VWF:Ag ratios with AS severity

**Figure 6:**
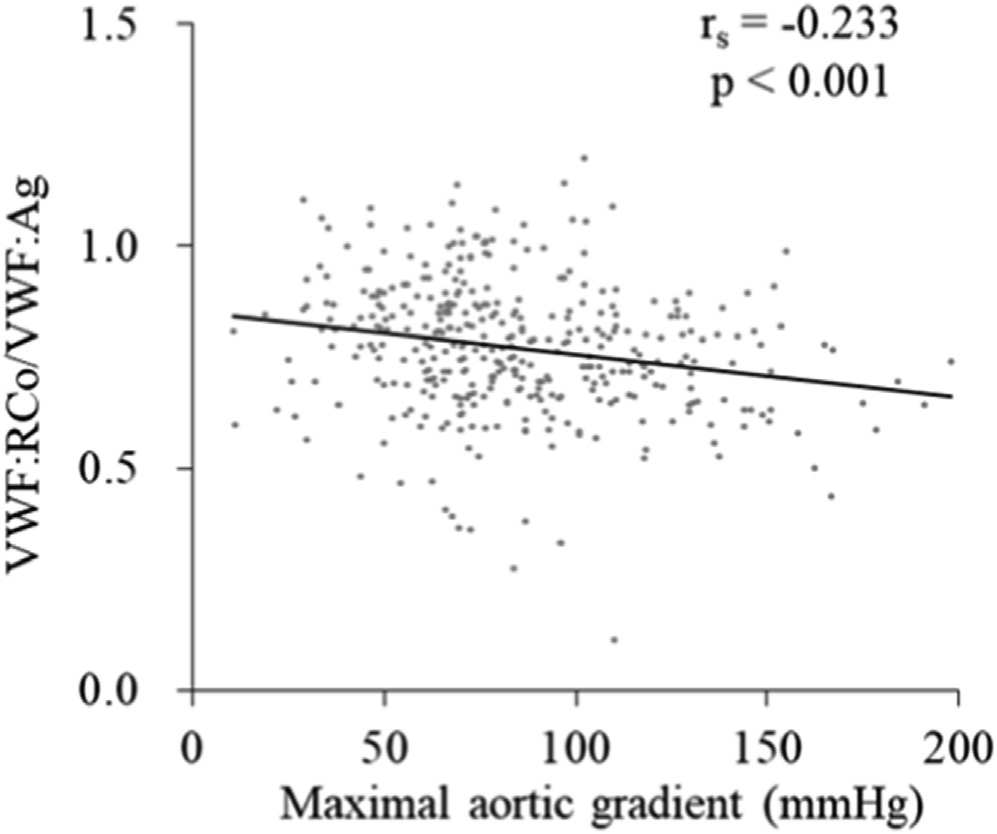
Correlation of von Willebrand factor Ristocetin co-factor activity/von Willebrand factor antigen with the maximal pressure gradients through the aortic valve in patients with aortic stenosis. These were weakly correlated (r_s_ = −0.233, *P* < .001).

We evaluated the effects of transaortic valve maximal pressure gradients, a marker of the severity of AS, on VWF:RCo/VWF:Ag. We found that VWF:RCo/VWF:Ag weakly correlated with maximal pressure gradients (r_s_ = −0.233, *P* < .001), although the tilt angle was shallow (Figure 6), compared to that of VWF large multimer indices with this cohort (r_s_ = -0.495, *P* < .001, manuscript in preparation by Miura et al.) or to that shown previously by Tamura et al. [10] (r = −0.64, *P* < .0001).

### 3.5 Relationship between FVIII activity and VWF-related values

**Figure 7:**
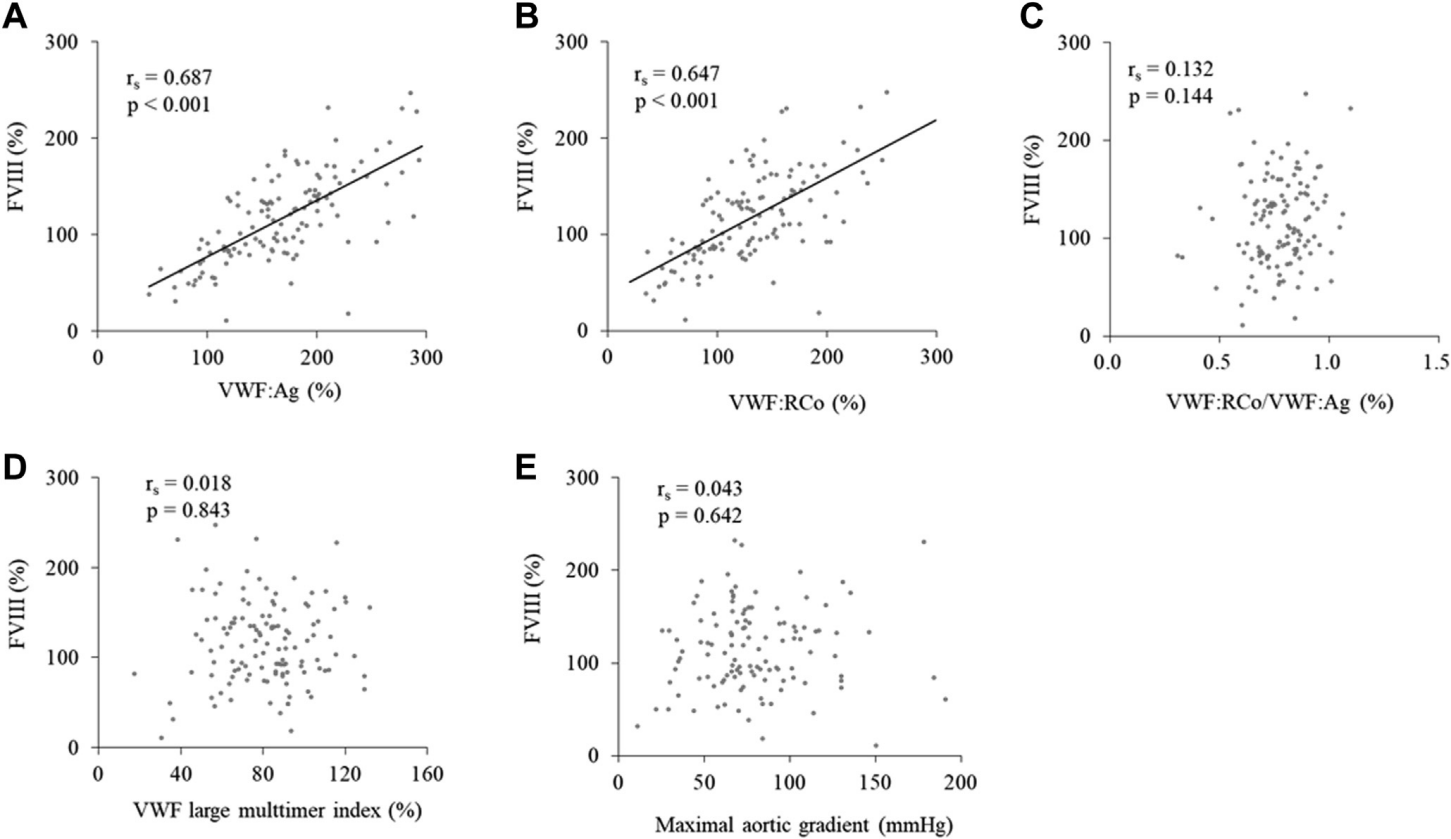
Correlation between factor (F) VIII activities and VWF:Ag (r_s_ = 0.687, *P* < .001) (A), VWF:RCo (r_s_ = 0.647, *P* < .001) (B), VWF:RCo/VWF:Ag (r_s_= 0.132, *P* = .14) (C), VWF large multimer indices (r_s_ = 0.018, *P* = .84) (D), or the transaortic valve maximal pressure gradients (r_s_ = 0.043, *P* = .64) (E). *n* = 123. VWF, von Willebrand factor; VWF:Ag, VWF antigen; VWF:RCo, VWF Ristocetin co-factor activity.

It is well known that FVIII forms a complex with VWF, which is required for stable existence of FVIII in blood while their molecular ratios are 50 to 100:1 [[22], [23], [24], [25], [26]]. Nevertheless, some studies have reported that binding of FVIII to VWF does not require VWF multimer formation [27,28], while others have reported that its binding is dependent on VWF multimer formation [29,30]. As a final set of analysis, we examined the relationship between FVIII activities and VWF-related values in 123 patients in this study. FVIII activities positively correlated with VWF:Ag (r_s_ = 0.687, *P* < .001; Figure 7A), and with VWF:RCo (r_s_ = 0.647, *P* < .001; Figure 7B), even when VWF:Ag and VWF:RCo increased up to 300%. In comparison, no significant correlations were detected between FVIII activities and VWF:RCo/VWF:Ag (r_s_ = 0.132, *P* = .14; Figure 7C), VWF large multimer indices (r_s_ = 0.018, *P* = .84; Figure 7D), or transaortic valve maximal pressure gradients (r_s_ = 0.043, *P* = .64; Figure 7E).

## 4 Discussion

In this study, we investigated a large number of patients with AS to evaluate the usefulness of VWF:RCo/VWF:Ag for the diagnosis of AS-induced AVWS. The gold standard diagnostic method for shear stress–induced AVWS is the VWF multimer analysis, although western blotting is difficult to perform for analyzing huge VWF multimers in hospital laboratories. Furthermore, a reference value for the analysis is lacking since it has seldomly been evaluated quantitatively. Thus, at the beginning of the study, we defined a reference value for the VWF multimer analysis that indicated loss of VWF large multimers. We here found that the VWF large multimer indices <80% may indicate AVWS for following reasons: (1) the indices of <80% exhibited an apparent reduction in the large multimers (Figure 1A); (2) 59.0% of patients with severe AS, while no patients with PAD or control patients except 2 patients exhibited the indices of <80% (Figure 1C–E). Thus, using the VWF large multimer indices <80% as the reference value, we evaluated VWF:RCo/VWF:Ag in this study.

VWF:RCo/VWF:Ag ratios moderately correlated with the VWF large multimer indices. Further, 71.2% of patients with VWF:RCo/VWF:Ag of <0.7 exhibited the VWF large multimer indices of <80%. When VWF:RCo/VWF:Ag ratio of <0.7 was used as a cut-off point, the sensitivity and specificity for VWF large multimer indices of <80% were 0.437 and 0.826, respectively. Thus, VWF:RCo/VWF:Ag of <0.7 may indicate loss of VWF large multimers with excellent specificity although its sensitivity is rather low.

When VWF:RCo/VWF:Ag of <0.7 is used for the diagnosis of AS-induced AVWS, we need to consider following: patients with PAD exhibited the VWF:RCo/VWF:Ag of around 0.8, which is close to 0.7; VWF:RCo/VWF:Ag ratios were correlated with the severity of AS although the tilt angle of the slope of the regression line was rather low, compared to that of VWF large multimer indices.

In the guidelines of the American Society of Hematology, the International Society on Thrombosis and Haemostasis, the National Hemophilia Foundation, and the World Federation of Hemophilia for the diagnosis of hereditary von Willebrand disease, a bleeding tendency and a decrease in VWF:RCo and/or VWF:Ag are essential criteria for its diagnosis while these are not included in the diagnosis of AVWS [20]. Although there are no clear diagnostic criteria for AVWS, several articles outline the following criteria for the diagnosis of AVWS [[31], [32], [33]]: a bleeding disorder without family and personal histories of von Willebrand disease; VWF:Ag, VWF:RCo/VWF:Ag, or VWF high molecular weight (large) multimers are reduced/absent. In our study, the VWF:Ag levels in patients with AS and control patients were rather high (168.4% ± 53.1% and 182.9% ± 56.2%, respectively). This is, at least partly, due to an age-dependent increase in VWF antigen levels [[34], [35], [36], [37]] since the patients with AS and control patients analyzed here had a mean age of 82 ± 7 years and 76 ± 5 years, respectively. Even if VWF:RCo becomes relatively low compared with VWF:Ag due to a loss of high molecular weight VWF multimers, the VWF:RCo of most of patients with AS remained >100%. However, AS is well known to accompany bleedings, especially gastrointestinal bleedings [7,38,39]. Typically, gastrointestinal bleeding is considered from the fragile angiodysplasia under a hemostatic disorder condition of AVWS [40]. Thus, the pathophysiology should be elucidated in a hemostatic condition with VWF:RCo around 100% in the presence of a loss of VWF large multimers *in vivo* and *in vitro*.

AVWS is caused by several cardiovascular diseases with high shear stress. PAD is caused by a stenosis or obstruction of an artery in the lower extremities. Thus, flow velocity is accelerated at the site of stenosis to some extent. However, no patients with the disease exhibited the VWF large multimer indices of <80% in our study. Thus, PAD may not be associated with AVWS. This could be due to relatively low flow velocity at the stenosis in a peripheral artery since more blood flows to other vessels that do not have a stenosis, which have lower resistance than the stenotic vessel.

Here, we demonstrated no correlation between FVIII activity and VWF large multimer indices or VWF:RCo/VWF:Ag ratios, suggesting that VWF multimer formation may not be necessary for FVIII binding and subsequent stable existence of FVIII. Further, we found that FVIII was positively correlated with VWF:Ag up to 300% in patients with severe AS, and only 7.3% (9 in 123) of patients exhibited FVIII levels <50% (Figure 7A). Thus, we consider that FVIII levels are not reduced in patients with AS.

This study has some limitations. First, this study did not analyze bleeding events. The relationship between bleeding events and VWF:RCo/VWF:Ag could not be clarified in this study, which is under evaluation in our cohort study. Second, some patients exhibiting high VWF:Ag or VWF:RCo over the upper limit of the automated coagulation analyzer used here were not evaluated here. This should be considered since it could cause potential bias. Third, although we investigated factors listed in the patient characteristics (Table 1), it cannot be excluded that yet unknown confounders affected the results.

In conclusion, VWF:RCo/VWF:Ag ratios <0.7 may indicate loss of VWF large multimers detected by the VWF multimer analysis with high specificity, but low sensitivity. Thus, we may use VWF:RCo/VWF:Ag ratios in patients with AS, when their ratios are <0.7, to monitor the loss of VWF large multimers during their clinical courses.
